# Resisting the Heat: Bacterial Disaggregases Rescue Cells From Devastating Protein Aggregation

**DOI:** 10.3389/fmolb.2021.681439

**Published:** 2021-05-04

**Authors:** Panagiotis Katikaridis, Valentin Bohl, Axel Mogk

**Affiliations:** Center for Molecular Biology of the Heidelberg University and German Cancer Research Center, DKFZ-ZMBH Alliance, Heidelberg, Germany

**Keywords:** AAA+ protein, Hsp100, ClpB, ClpG, protein disaggregation, thermotolerance

## Abstract

Bacteria as unicellular organisms are most directly exposed to changes in environmental growth conditions like temperature increase. Severe heat stress causes massive protein misfolding and aggregation resulting in loss of essential proteins. To ensure survival and rapid growth resume during recovery periods bacteria are equipped with cellular disaggregases, which solubilize and reactivate aggregated proteins. These disaggregases are members of the Hsp100/AAA+ protein family, utilizing the energy derived from ATP hydrolysis to extract misfolded proteins from aggregates via a threading activity. Here, we describe the two best characterized bacterial Hsp100/AAA+ disaggregases, ClpB and ClpG, and compare their mechanisms and regulatory modes. The widespread ClpB disaggregase requires cooperation with an Hsp70 partner chaperone, which targets ClpB to protein aggregates. Furthermore, Hsp70 activates ClpB by shifting positions of regulatory ClpB M-domains from a repressed to a derepressed state. ClpB activity remains tightly controlled during the disaggregation process and high ClpB activity states are likely restricted to initial substrate engagement. The recently identified ClpG (ClpK) disaggregase functions autonomously and its activity is primarily controlled by substrate interaction. ClpG provides enhanced heat resistance to selected bacteria including pathogens by acting as a more powerful disaggregase. This disaggregase expansion reflects an adaption of bacteria to extreme temperatures experienced during thermal based sterilization procedures applied in food industry and medicine. Genes encoding for ClpG are transmissible by horizontal transfer, allowing for rapid spreading of extreme bacterial heat resistance and posing a threat to modern food production.

## Severe Heat Stress: the Loss of Essential Proteins by Aggregation Calls for Reactivating Disaggregases

**FIGURE 1:**
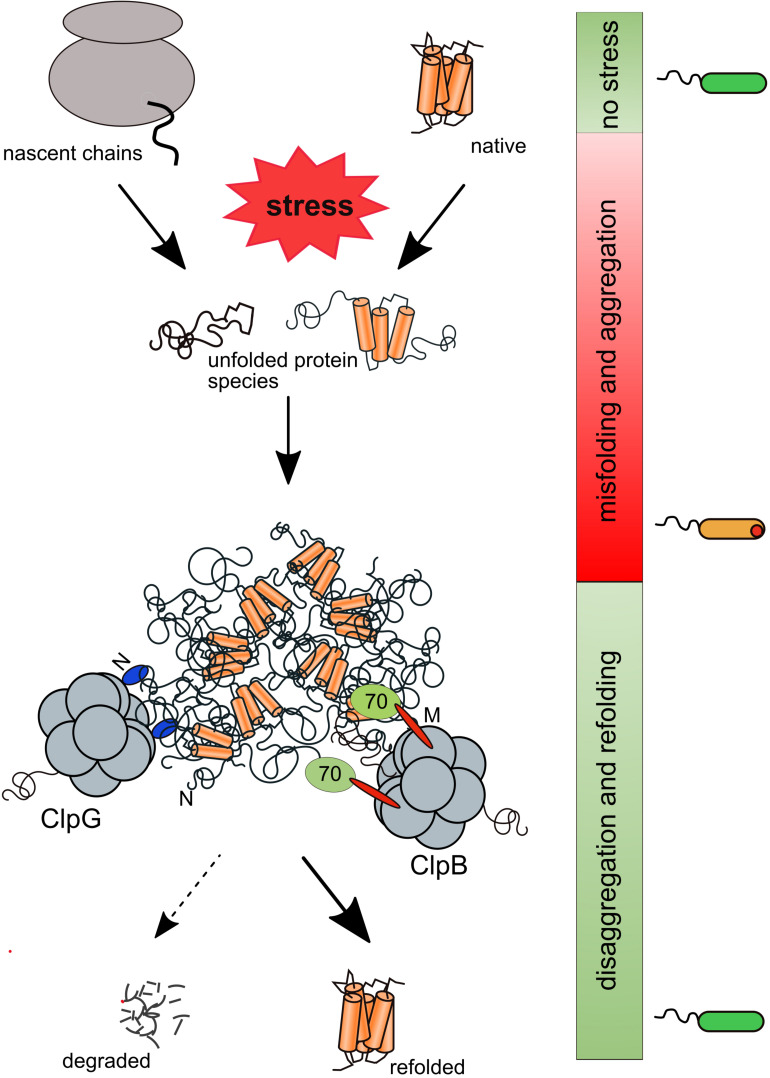
Rescue of aggregated proteins by the bacterial ClpB/Hsp70 and ClpG disaggregation systems. Stress conditions cause misfolding and aggregation of nascent polypeptides and native proteins. Protein aggregates include essential proteins and are deposited at the cell poles of bacteria. Aggregated proteins are solubilized and predominantly reactivated by the Hsp70-dependent ClpB disaggregase or ClpG, which functions autonomously. ClpB binds via its M-domains (M) to aggregate-bound Hsp70 while ClpG is directly binding to protein aggregates via a specific N-terminal domain (N).

Protein quality control (PQC) systems are composed of molecular chaperones and ATP-dependent proteases and maintain protein homeostasis by preventing the accumulation of misfolded proteins through refolding and degrading activities. Environmental stress conditions like heat shock that cause enhanced misfolding of newly synthesized or preexisting proteins trigger stress responses, which lead to an increase in the levels of PQC components, thereby enabling cells to deal with the increased load for the proteostasis network. However, severe stress conditions lead to a massive accumulation of misfolded proteins overwhelming the protective capacity of chaperones and proteases. Intermolecular, hydrophobic interactions of stress-induced misfolded protein species ultimately lead to the formation of protein aggregates (Figure 1). Protein aggregates are deposited at the cell poles of bacteria involving a passive process that is mainly driven by nucleoid occlusion (Winkler et al., 2010). While protein aggregates have been associated with bacterial cell death, there is currently no evidence that stress-generated protein aggregates exert a toxic activity. Along this line, bacteria can harbor large inclusion bodies formed by overproduced proteins that fail to fold properly, without showing detrimental effects on growth. So why is protein aggregation becoming a critical factor for bacterial survival during severe heat stress? The analysis of heat-induced protein aggregates revealed the presence of various proteins playing essential roles in diverse housekeeping processes including cell division (e.g., FtsZ), transcription (e.g., RNA polymerase) and translation (e.g., EF-G) (Mogk et al., 1999; Pu et al., 2019). The degree of protein aggregation is strongly increasing with stress severity. It is thus the loss of essential proteins caused by aggregation that limits cellular viability at high temperatures. Similarly, protein aggregates that form in bacterial persister cells are associated with their dormant state (Pu et al., 2019).

The loss of essential proteins during severe stress conditions creates a need for cellular disaggregases, which revert the aggregation process and rescue the lost proteins. Disaggregases thereby provide thermotolerance or heat resistance to cells. Members of the ATP-dependent Hsp100/AAA+ protein family play the central role in this disaggregation process. Hsp100 proteins come in two flavors. Most family members (e.g., ClpA, ClpC, ClpX) associate with peptidases (e.g., ClpP) to form bacterial proteasomes acting in regulatory and general proteolysis (Olivares et al., 2018). Some Hsp100 chaperones do not interact with peptidases and primarily direct substrates toward refolding pathways. The two best characterized bacterial disaggregases, ClpB and ClpG, belong to the latter category underlining the point that bacterial survival during extreme stress conditions demands for the reactivation of aggregated proteins (Figure 1). Accordingly, in yeast cells, aggregated proteins are not directed toward degradation pathways upon solubilization by Hsp104, the yeast homolog of ClpB, but are quantitatively refolded (Wallace et al., 2015). Furthermore, an engineered ClpB variant, which interacts with ClpP and targets aggregated proteins to degradation, does not provide heat resistance (Weibezahn et al., 2004).

In this review, we describe the physiology and molecular mechanisms of the bacterial ClpB and ClpG disaggregases. We refer to common principles of the disaggregases and point to specific differences in their modes of action and regulation. Finally, we describe the interplay of both disaggregases when coexisting in bacterial cells and how man-made stress conditions generate an essential need for the novel ClpG disaggregase.

## Basic Structural and Mechanistic Features of Hsp100/AAA+ Disaggregases

The bacterial disaggregases ClpB and ClpG are members of the Hsp100/AAA+ protein family. Hsp100/AAA+ proteins harbor a conserved AAA domain that includes conserved Walker A and B motifs for ATP binding and hydrolysis. The AAA domain(s) also mediates oligomerization, usually into a homohexameric ring that includes a central channel. Functional specificity of Hsp100 proteins is gained by the presence of extra domains, which are either fused to or inserted into the AAA domain. These additional domains are not part of the Hsp100 ring structure but they are exposed and function as binding platforms for cooperating proteins (adaptors) and for substrates. ClpB and ClpG disaggregases harbor two AAA domains (AAA1, AAA), which form separate ATPase rings. The functionality of both rings is crucial for efficient protein disaggregation (Schlee et al., 2001; Watanabe et al., 2002; Mogk et al., 2003; Katikaridis et al., 2021).

**FIGURE 2:**
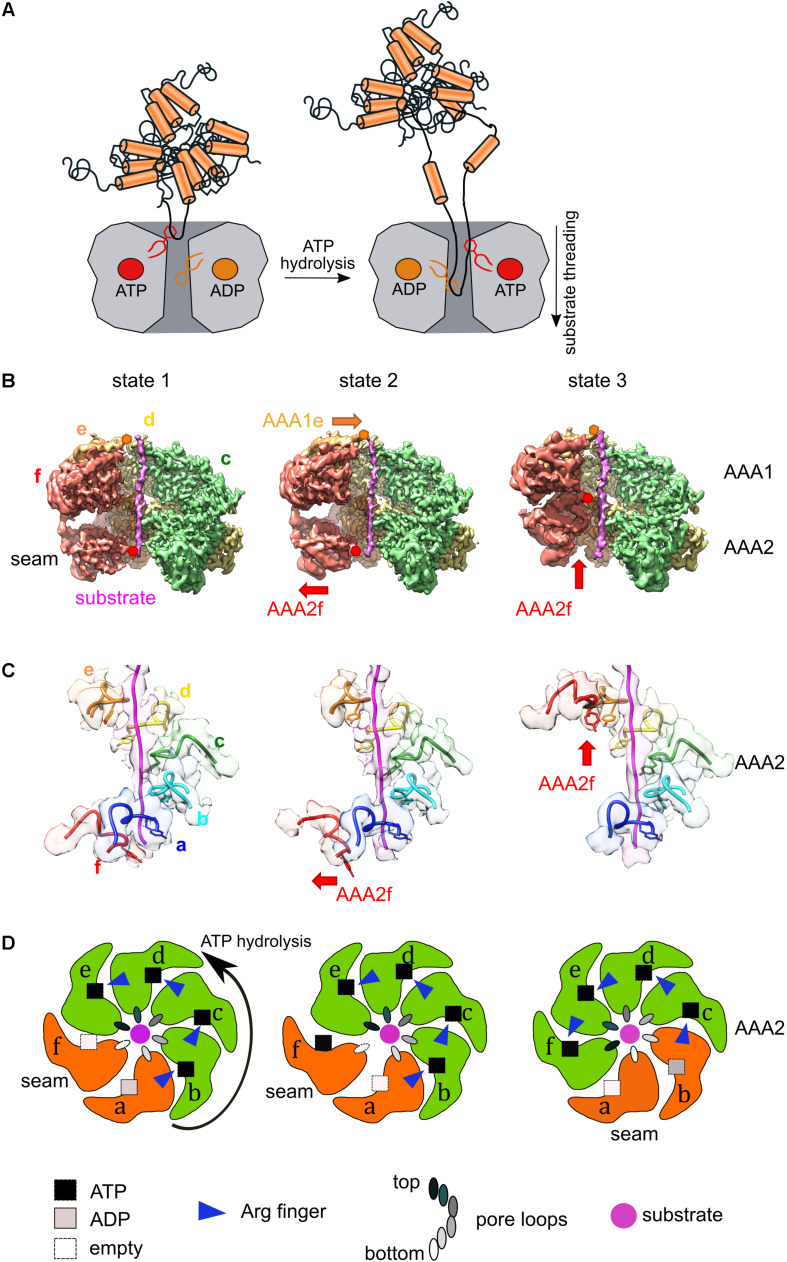
Coupling of ATPase and threading activities of Hsp100/AAA+ proteins. **(A)** Pore-located aromatic residues positioned at the central channel of Hsp100 hexamers bind protein substrates and pull linear or looped segments into the translocation channel upon ATP hydrolysis. **(B)** Cut views of the cryo-EM maps of three states of substrate (casein)-bound ClpB-K476C, which is an activated M-domain mutant. Conformational changes of protomers AAA1e and AAA2f are shown (state 2). Substrate engagement in the AAA1 ring is linked to substrate dissociation in the AAA2 ring. Subunit AAAf shows a large conformational rearrangement upon its activation in state 3. **(C)** Pore loop interactions of the ClpB-K476C AAA2 ring with the substrate casein in the three structural states. Panels **(B,C)** modified from Deville et al. (2019). **(D)** Schematic representation of the three functional states of the AAA2 ring of activated ClpB-K476C. Nucleotide states (ATP, ADP, empty), engaged arginine fingers and the positions of pore loops of the individual subunits are indicated. Active AAA2 subunits are shown in green, inactive ones in orange. ATP hydrolysis proceeds in a counterclockwise manner. Activation of the seam subunit f is linked to ATP hydrolysis in subunit b, leading to its inactivation.

Hsp100 proteins act in protein unfolding, protein disaggregation and protein complex disassembly processes, in which they generate a mechanical force fueled by ATP hydrolysis. A threading activity is key for the molecular functions of Hsp100 proteins. Threading involves the application of a pulling force and is applied by conserved aromatic residues, which are crucial for Hsp100 activities (Lum et al., 2004; Schlieker et al., 2004; Weibezahn et al., 2004) (Figure 2A). The aromatic side chains are part of mobile loops that are positioned at the central translocation channel. These pore loops directly bind protein substrates and change positions during the Hsp100 ATPase cycle thereby threading the bound substrate through the translocation channel (Puchades et al., 2020). Threading can be initiated at N- or C-terminal ends of substrate proteins, but also at internal segments, leading to threading of either linear or looped polypeptides, respectively (Hoskins et al., 2002). It is rather unlikely that N- or C-termini of aggregated proteins are accessible for recognition by bacterial disaggregases, which will therefore preferentially act on internal sequence stretches. Indeed, ClpB can thread loop structures and efficiently solubilizes protein aggregates that only offer internal segments for processing (Haslberger et al., 2008). The threading of substrate loops involves the translocation of two polypeptide arms and ClpB can switch between two-arms and single-arm translocation during the disaggregation reaction (Avellaneda et al., 2020).

The pulling forces that are applied by Hsp100/AAA+ proteins can be very high and allow for the unfolding of tightly folded protein domains (Weber-Ban et al., 1999). An activated ClpB mutant exerts forces of more than 50 pN at speeds of more than 500 residues per second (Avellaneda et al., 2020). This qualifies ClpB as one of the strongest unfolding machines characterized to date and also indicates the need to tightly control ClpB unfolding power and substrate specificity to avoid uncontrolled and detrimental unfolding events.

Major breakthroughs in understanding the mechanism of substrate threading by Hsp100/AAA+ proteins and its coupling to the ATPase cycle have been recently achieved by determining their hexameric structures by cryo electron microscopy (cryo EM). Here, we focus on the structures of the ClpB disaggregase and its yeast homolog Hsp104, which both share common features with other AAA+ proteins, pointing to a conserved mechanism of ATP hydrolysis and substrate threading (Deville et al., 2017, 2019; Gates et al., 2017; Yu et al., 2018; Rizo et al., 2019). ClpB/Hsp104 form asymmetric hexameric assemblies, which resemble shallow spirals. The asymmetric arrangement of the subunits is well illustrated by the positions of the substrate contacting pore loops: they are not arranged at one level of the translocation channel but form a spiral staircase that almost entirely spans the ATPase rings (Figures 2B,C). One of the subunits, termed the seam subunit, is most displaced from the central ring axis (Figure 2B). It is typically less well resolved in the cryo EM reconstructions indicating increased mobility.

ClpB/Hsp104 subunits exist in different activity states despite having identical sequences. An active subunit is capable of hydrolyzing ATP. It has ATP bound at the active center and the ATP is additionally contacted by an allosteric arginine residue (arginine finger) from the clockwise subunit. An inactive subunit does not receive an arginine finger and has either no nucleotide or ADP bound. In most ClpB/Hsp104 structures three to five AAA subunits are active, while one to three represent inactive states. Positions of pore loops are determined by the activity states of the respective AAA domains. The pore loop of an inactive subunit is positioned at the bottom of the spiral staircase and moves to the top position upon activation (Figures 2B–D). The order of active and inactive subunits in the hexameric assemblies suggests that ATP hydrolysis propels around the AAA ring in a counterclockwise manner (Figure 2D). The determination of multiple ClpB/Hsp104 structures and their grouping into a specific order imply a sequential firing mode. ATP hydrolysis in active subunits and the reactivation of previously inactive subunits are coupled to large movements of AAA subunits during the ATPase cycle, which in turn alter the position of pore loops. Thus, the ATPase cycle propels the cycling of pore-located from the top position of the spiral staircase to the bottom and back to the top (Figures 2B–D). The ClpB reaction cycle demands for large scale transitions of individual subunits, which is consistent with high-speed atomic force microscopy, revealing the existence of diverse dynamic states of ClpB hexamers (Uchihashi et al., 2018; Inoue et al., 2021).

The disaggregases ClpB and ClpG harbor two ATPase rings raising the question about their specific contributions to substrate threading and their coordination during the threading cycle. While both ATPase rings are crucial for protein disaggregation, biochemical and structural data point to functional differences and qualify the AAA2 ring as main threading motor (Deville et al., 2019). Accordingly, the pore loop2 of the AAA2 subunit is essential for substrate threading, while pore loop1 mutants still retain substantial disaggregation activities (Deville et al., 2019; Katikaridis et al., 2021). A recent study on the Hsp100 member ClpA indicates that the AAA1 ring enhances substrate grip, thereby preventing backsliding (Kotamarthi et al., 2020). Notably, the two ATPase rings of ClpB do not seem to work synchronously but their ATPase and threading cycles are shifted by one phase. This means that substrate engagement by a pore loop of an AAA1 subunit is linked to substrate dissociation in a neighboring AAA2 subunit (Figure 2B) (Deville et al., 2019).

A sequential mode of ATP hydrolysis is predicted to propel substrate translocation in small, discrete steps of two residues per hydrolyzed ATP. Similar models have been proposed for other Hsp100/AAA+ proteins (Gates and Martin, 2020; Puchades et al., 2020), suggesting a conserved working principle. Still, one needs to consider that cryo EM structures represent snapshots and are typically derived from states that cannot or only slowly hydrolyze ATP. The grouping of multiple Hsp100 structures into a certain order is on the one hand meaningful and important, however, it also poses a challenge as alternative ways of structure conversions cannot be ruled out. Accordingly, Hsp104 mutant hexamers captured in the ATP state can adopt diverse ring conformations, which had been associated with distinct nucleotide states before (Lee et al., 2019). The cryo EM structures and the derived models for ATP hydrolysis and substrate threading are therefore in need of biochemical analysis. Of note, the available biochemical data are diverse and in parts support a sequential ATP hydrolysis and threading mechanism, yet they also raise issues that still need to be addressed.

A sequential model is supported by poisoning experiments, which rely on the mixing of ATPase deficient and wild type subunits leading to the formation of mixed hexamers. Determining ATPase and threading activities of mixed oligomers formed upon combining mutant and wild type subunits at different ratios various revealed that the incorporation of a single ATPase deficient subunit is sufficient to abrogate ClpB disaggregation activity (Kummer et al., 2016; Deville et al., 2019). This indicates that a highly coordinated mode of ATP hydrolysis is required for function. On the other hand, step sizes of ClpB substrate threading determined by single molecule experiments do not reconcile with a threading mode based on continuous small steps. An activated ClpB threads up to 28 substrate residues in single bursts before shortly pausing substrate translocation (Avellaneda et al., 2020). This observation could be explained by consecutive, yet very rapid firing of all subunits. Resetting the ClpB ring might than demand for exchange of ADP for ATP in all subunits to initiate a new ATPase and threading cycle (Avellaneda et al., 2020). Consistent with such possibility, ADP release has been described as rate-limiting step in the ATPase cycle of Hsp104 (Ye et al., 2020). Further biochemical studies that also analyze the ClpB ring dynamics in absence and presence of substrate and in its diverse activity states (see below) will be necessary to dissect how the ClpB ATPase and threading cycles are orchestrated.

## ClpB: A Widespread and Partner Controlled Disaggregase

**FIGURE 3:**
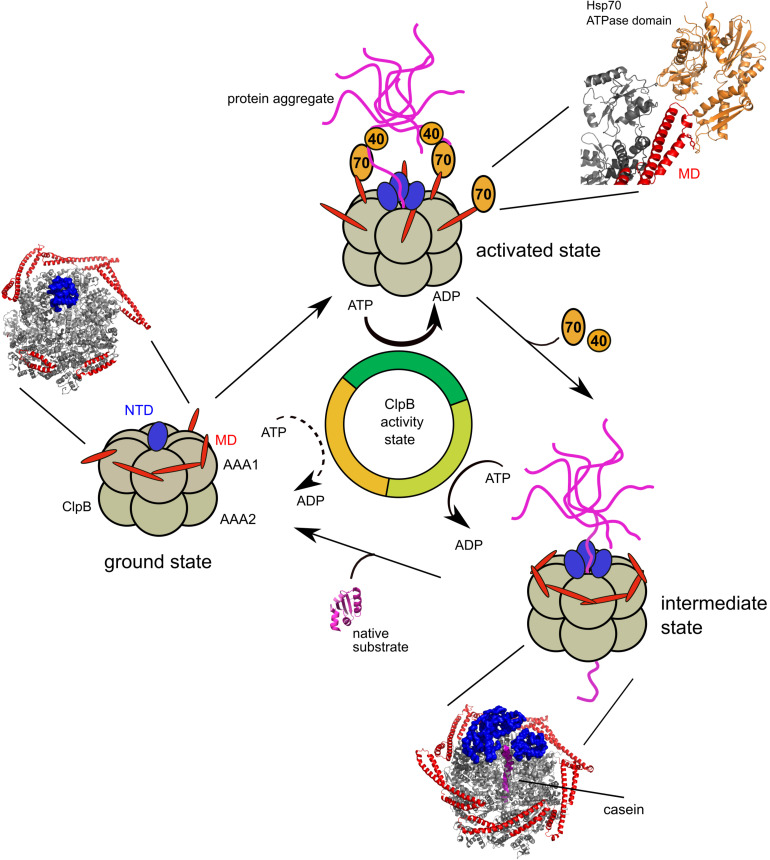
Mechanism of ClpB activity control by Hsp70 and substrate interactions. ClpB in its ground state exhibits low ATPase activity. M-domains are existing in horizontal and tilted positions, which are predicted to interchange rapidly. The pore entrance of the translocation channel is blocked by an N-terminal domain (NTD). Recruitment of ClpB to protein aggregates by Hsp70 (DnaK) and its co-chaperone Hsp40 (DnaJ) stabilizes M-domains in tilted conformations, causing ClpB activation. Substrate engagement leads to high ATP hydrolysis rates. NTDs contribute to substrate binding and eventually facilitate substrate entry into the channel. Upon dissociation of Hsp40/Hsp70 ClpB M-domains rearrange into horizontal positions lowering ClpB ATPase and threading activity (intermediate state). Protein substrates refold after completion of translocation and ClpB converts back to the ground state. Cryo-EM structures of *E. coli* ClpB wild type (±substrate) and a model of the Hsp70 (DnaK) ATPase domain bound to a ClpB M-domain are shown.

ClpB represents the most widespread bacterial disaggregase that exists in most Gram-negative and Gram-positive bacteria. Eukaryotic ClpB homologs are found in the cytosol/nucleus as well as mitochondria and chloroplasts of plants and unicellular eukaryotes, including *S. cerevisiae*. *clpB* deletion mutants are typically linked to a loss of thermotolerance, qualifying ClpB as the dominating bacterial disaggregase (Squires et al., 1991; Chastanet et al., 2004; Frees et al., 2004; Tripathi et al., 2020). Notably, ClpB on its own does not exert disaggregation activity but crucially relies on cooperation with an Hsp70 partner chaperone, which functions as targeting factor and allosteric activator of ClpB (Figure 3).

ATP-dependent Hsp70 (bacterial DnaK) represents a highly abundant and major chaperone of bacteria with crucial functions in *de novo* protein folding, the refolding of misfolded proteins and protein targeting (Rosenzweig et al., 2019). Hsp70 itself acts in concert with co-chaperones, including J-domain proteins (bacterial DnaJ, CbpA) and nucleotide exchange factors (bacterial GrpE) (Mayer and Kityk, 2015). DnaJ targets DnaK to diverse substrates and couples substrate delivery with stimulation of DnaK ATPase activity, allowing for tight binding of substrate proteins. GrpE binding causes dissociation of nucleotide (ADP) from DnaK. This resets the DnaK ATPase cycle and allows for substrate dissociation upon ATP rebinding. In the context of protein disaggregation, DnaJ targets DnaK to the surface of protein aggregates (Carrio and Villaverde, 2005; Acebron et al., 2009; Winkler et al., 2012). The coating of the aggregate surface by DnaK provides specificity for reactivating ClpB and prevents binding of Hsp100/AAA+ proteins (e.g., ClpA/ClpC) that cooperate with peptidases (e.g., ClpP) (Haslberger et al., 2008). DnaK thereby impacts triage decision and ensures that aggregated proteins will be primarily targeted to refolding pathways. A single DnaK partner is not sufficient for ClpB recruitment, but at least two DnaK proteins are required (Seyffer et al., 2012). This high density of substrate-bound DnaK likely provides specificity for exclusive targeting of ClpB to protein aggregates, while avoiding disaggregase recruitment to, e.g., nascent polypeptide chains (Figure 3).

A limited, Hsp70-independent disaggregation activity of ClpB/Hsp104 has been reported in presence of ATP/ATPγS mixtures (Doyle et al., 2007a,b; Hoskins et al., 2009). The gained disaggregation activity is, however, limited and also dependent on the aggregated model protein. Furthermore, this mixed nucleotide state is non-physiological and cannot enable ClpB to work autonomously *in vivo*. Accordingly, *E. coli dnaK* mutant cells are deficient in protein disaggregation, underlining the crucial function of Hsp70 (DnaK) as targeting factor (Mogk et al., 1999). Similarly, activated ClpB/Hsp104 M-domain mutants, which do no longer require Hsp70 for stimulation of ATP hydrolysis, exhibit only negligible disaggregation activity and remain dependent on cooperating Hsp70 (Jackrel et al., 2014; Kummer et al., 2016).

The targeting function of Hsp70 relies on direct physical contacts with ClpB and involves the binding of the Hsp70 ATPase domain to the central regulatory M-domain of the disaggregase (Seyffer et al., 2012; Lee et al., 2013; Rosenzweig et al., 2013). Notably, the substrate binding domain of Hsp70 is also required for partnering with ClpB, suggesting that this domain also contacts the disaggregase (Fernandez-Higuero et al., 2018). Its role as interaction platform for Hsp70 explains the essential function of the M-domain for protein disaggregation (Kedzierska et al., 2003; Mogk et al., 2003). M-domains also fulfill a crucial regulatory function and tightly control ClpB ATPase and threading activities (Haslberger et al., 2007; Oguchi et al., 2012), thereby enabling the timely activation of the disaggregase upon recruitment to the aggregate surface.

The M-domain forms a coiled-coil structure composed of two wings, termed motif1 and motif2 (Lee et al., 2003). Initial structure determinations of ClpB hexamers, based on negative staining and applying symmetry constraints, revealed that M-domains exist in horizontal and tilted structural states, which are linked to low and high activity states, respectively (Carroni et al., 2014). Neighboring, horizontal M-domains interact in a head-to-tail manner via their motifs 1 and 2. These interactions stabilize M-domains in a horizontal state, leading to the formation of a repressing belt, which engulfs the AAA1 ring. This restricts the conformational dynamics of the ATPase ring, which is necessary for high ATPase and threading activities (Heuck et al., 2016). Recent findings indicate that the M-domain of Hsp104 confines ADP release, which might represent a rate-limiting step in the ClpB/Hsp104 ATPase cycle (Ye et al., 2020). M-domain interactions are broken in tilted M-domains, relieving the ATPase ring and strongly increasing ATP hydrolysis rates. The initial structural picture of M-domain organizations was recently refined by asymmetric reconstruction of ClpB/Hsp104 hexamers based on cryo EM. Here, M-domains are visible to varying degrees, indicating the co-existence of horizontal and tilted M-domains in a single ClpB/Hsp104 hexamer (Figure 3) (Deville et al., 2017, 2019; Gates et al., 2017). Horizontal M-domains are located next to each other, due to their reciprocal stabilizing interactions. The determined cryo EM structures represent one snapshot of the asymmetric ClpB hexamers and M-domain states should not be taken static. Indeed, it was recently demonstrated that M-domains switch their conformations on a microsecond time scale (Mazal et al., 2019). This rapid exchange ensures that ClpB permanently exists in an Hsp70-activatable state, while overall remaining repressed in absence of the Hsp70 partner.

Hsp70 binds to motif2 of the M-domain (Seyffer et al., 2012; Rosenzweig et al., 2013), which is only accessible in the tilted conformation but inaccessible in the horizontal state due to its interaction with a neighboring M-domain. The binding of Hsp70 stabilizes the M-domain in a tilted state thereby ultimately leading to ClpB activation (Figure 3). M-domains of activated ClpB/Hsp104 mutants predominantly exist in the tilted state and enhance ATPase activity by facilitating ADP release (Carroni et al., 2014; Mazal et al., 2019; Ye et al., 2020). Similarly, Hsp70 binding to M-domains reduces the inhibitory effects of ADP on ClpB disaggregation activity (Klosowska et al., 2016). Next to Hsp70, substrate binding plays a second important role in triggering ClpB activation and highest ClpB ATP hydrolysis rates are only achieved in the presence of both, the Hsp70 partner and substrate (Kummer et al., 2016; Fernandez-Higuero et al., 2018; Deville et al., 2019). This suggests that full ClpB activation is achieved upon substrate transfer from Hsp70 to ClpB. Whether a direct transfer takes place or whether ClpB binds to a different segment of the aggregated protein located in close vicinity is unknown. *In vitro*, ClpB is capable of actively displacing a substrate from Hsp70 by applying a pulling force (Rosenzweig et al., 2013).

The activated state of ClpB likely exists only transiently and the disaggregase turns into a partially activated state during the disaggregation reaction (Figure 3). This model is supported by biochemical findings showing that ClpB has a reduced unfolding power as compared to other Hsp100 family members (Haslberger et al., 2008). Thus, ClpB cannot unfold stably folded domains of proteins trapped in a protein aggregate. A transient activation of ClpB is also supported by the cryo EM structure of substrate (casein) bound ClpB, which shows the formation of a complete M-domain ring surrounding the ClpB hexamer (Deville et al., 2017). From these data, a model can be derived, which predicts that the fully activated state of ClpB is short-lived and limited to the initial stage of substrate engagement. Hsp70 will dissociate from ClpB upon substrate transfer and the reorganization of M-domains will convert ClpB from a high to an intermediate activity state. Upon completion of substrate threading ClpB will switch back to its ground state, which exhibits low activity yet is available for Hsp70 binding and activation (Figure 3). The essential need to tightly control ClpB activity is apparent from activated M-domain mutants, which exhibit high ATPase and unfolding activities in the absence of Hsp70. While these mutants still rely on Hsp70 for protein aggregate binding, their persistent activation causes severe cellular toxicity, likely by acting on essential proteins and causing their inactivation through unfolding events (Oguchi et al., 2012; Lipinska et al., 2013).

While the role of the M-domains in controlling ClpB activity and function in protein disaggregation is well characterized, the function of the N-terminal domain (NTD) remained poorly understood for a long time. The NTD is not essential for protein disaggregation and its deletion can reduce but also enhance ClpB disaggregation activity in a substrate specific manner (Beinker et al., 2002; Mogk et al., 2003; Barnett et al., 2005). The ClpB NTD harbors a hydrophobic groove that binds substrates and its differing contributions to protein aggregate binding might explain the diverse consequences of NTD deletion on disaggregation function. Recent reports illustrate a role of the NTD in regulating ClpB activity. A cryo EM model of ATP-bound *E. coli* ClpB shows a single NTD that is located on top of the central translocation channel, sealing the pore entrance (Figure 3) (Deville et al., 2017; Tripathi et al., 2018). The substrate binding groove of the NTD remains accessible in this state providing a pathway for substrate-induced ClpB activation. Such model predicts that initial substrate binding to the plugging NTD triggers a conformational rearrangement that makes the central channel accessible for substrate interaction and threading. NTD mutants, which are deficient in substrate binding due to mutations in the hydrophobic groove, exhibit a much stronger defect in protein disaggregation as compared to a complete NTD deletion mutant (Rosenzweig et al., 2015). Preventing substrate binding to the NTD thus causes a dominant inhibition of ClpB. In agreement with a substrate-induced movement of the plugging NTD, cryo EM structures of substrate casein bound ClpB revealed that the translocation channel is accessible and extended at the entry site by three NTDs, which contact casein and eventually help to correctly position the substrate for channel insertion (Deville et al., 2017; Rizo et al., 2019).

## ClpG: A Novel, Standalone Disaggregase Provides Superior Heat Resistance

Heat shock conditions in nature will predominantly involve temperature gradients with an upper temperature limit of 50–55°C. The activity of the canonical ClpB disaggregase evolved to ensure bacterial survival under these stress regimes. Furthermore, temperature gradients enable for cellular adaptation through the induction of stress responses, which lead to an increase in ClpB and Hsp70 levels, thereby increasing disaggregation capacity. Increased expression upon heat shock is also observed for other Hsp100 proteins including ClpC, ClpE, and ClpL, implying crucial functions in cellular PQC (Gerth et al., 2004; Li et al., 2011). However, not all family members are increasingly produced at elevated temperatures, such as ClpX (Gerth et al., 2004) or ClpG (Lee et al., 2018).

Bacteria face new forms of heat stress in the modern industrial world. These include thermal sterilization protocols applied in modern food production and hospitals to strongly reduce the numbers of contaminating, potentially harmful microorganisms. For instance, during Holder pasteurization milk is heated to 62.5°C for 30 min. Thermization is frequently used during cheese production and involves heating to 65°C for at least 15 s (Peng et al., 2013). These man-made stress applications will kill most bacteria. They do not allow for bacterial adaptation as the temperature upshift happens abruptly and its severity will trigger aggregation of crucial components of the transcription and translation machineries, making a cellular response impossible. However, bacteria seem to increasingly gain the ability to withstand the thermal sterilization protocols. Extremely heat-tolerant *E. coli* strains have been isolated from food factories (2% of all strains) (Dlusskaya et al., 2011; Li and Ganzle, 2016; Mercer et al., 2017). Similarly, *Klebsiella pneumoniae* pathogens exhibiting enhanced heat resistance were able to persist in the hospital environment (Bojer et al., 2010).

Extreme bacterial heat resistance is associated with a new member of the Hsp100/AAA+ protein family: ClpG (also termed ClpK) (Bojer et al., 2010, 2013; Lee et al., 2018). ClpG is present in selected Gram-negative bacteria including major pathogens like *Pseudomonas aeruginosa, Salmonella enterica, Enterobacter* sp. among others. The *clpG* gene is located on a gene cluster termed LHR (locus of heat resistance) or TLPQC (transmissible locus for PQC) (Lee et al., 2015; Mercer et al., 2015; Boll et al., 2017). The gene cluster comprises additional PQC components including chaperones (e.g., small heat shock proteins), proteases (e.g., FtsH) and factors involved in oxidative stress response (e.g., Thioredoxin). Importantly, the cluster is present on mobile genomic islands or on conjugative plasmids and can be horizontally transferred to other bacteria. This defines ClpG as novel virulence and persistence factor as it enhances bacterial fitness and enables pathogens to withstand sterilization procedures as described above. Deleting the *clpG* gene from the LHR/TLPQC causes the loss of extreme heat resistance, defining it as crucial core component (Bojer et al., 2011). However, sole expression of *clpG* in heat sensitive bacteria does not confer extreme heat resistance, indicating that additional LHR/TLPQC factors are involved in this process (Mercer et al., 2017). Growth conditions that mimic industrial food production increase the fraction of bacterial strains harboring ClpG. This underlines that man-made stress conditions exert a selective pressure favoring growth of *clpG* harboring bacteria and enhancing *clpG* spreading in bacterial populations (Mercer et al., 2015; Marti et al., 2016; Boll et al., 2017). Indeed, ClpG has been meanwhile identified in commensal and various clinical *E. coli* strains (Ma et al., 2020; Kamal et al., 2021).

The molecular basis of ClpG-mediated heat resistance was unraveled by showing that it acts as disaggregase. In contrast to ClpB, ClpG functions as standalone disaggregase and does not require assistance by partner proteins (Lee et al., 2018). *In vitro* ClpG disaggregation activity is similar or higher as compared to the canonical ClpB/Hsp70 disaggregation system.

**FIGURE 4:**
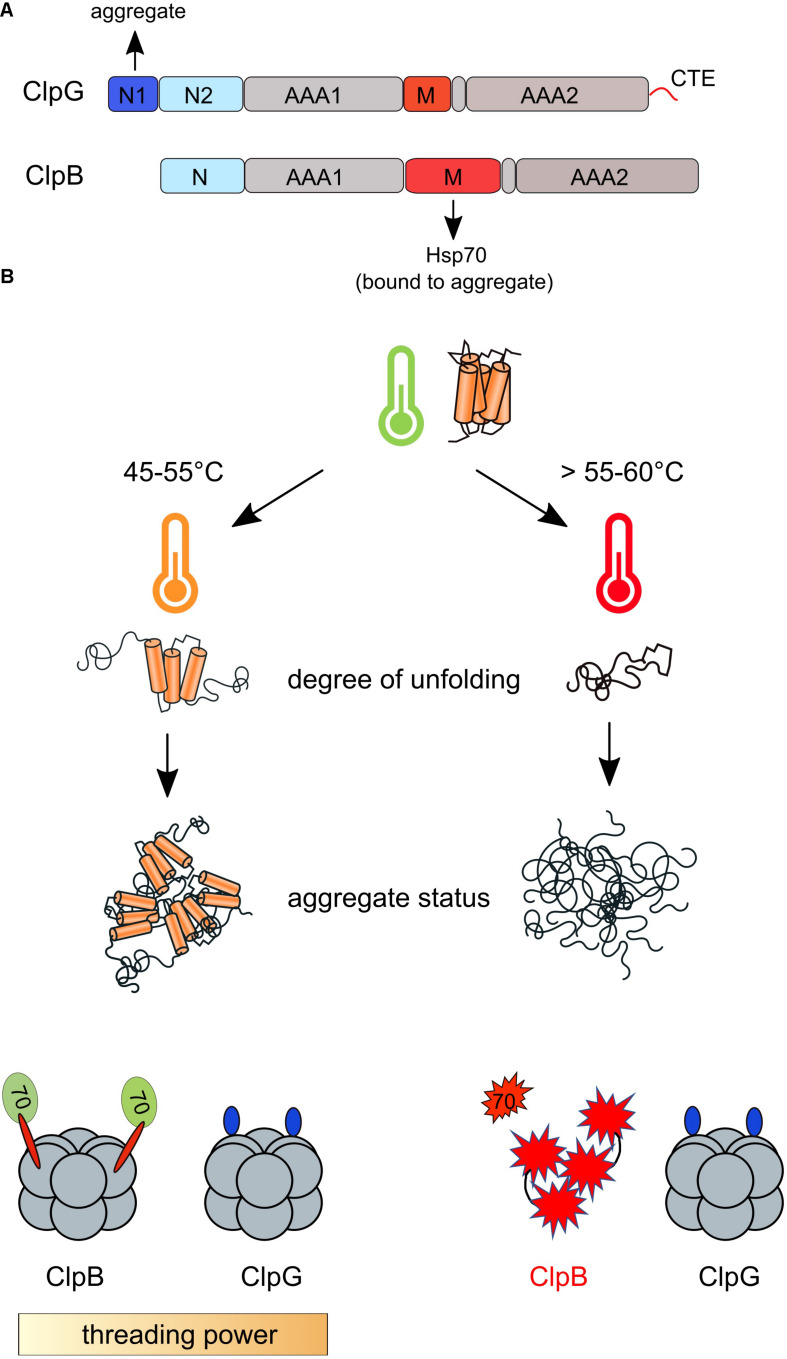
Contributions of ClpB/Hsp70 and ClpG disaggregases during physiological and extreme heat shock. **(A)** Domain organizations of ClpB and ClpG. Both disaggregases harbor two AAA domains (AAA1, AAA2), an M-domain and a homologous N-terminal domain (ClpB N, ClpG N2). ClpG harbors a specific N1 domain and a C-terminal extension (CTE). The ClpG N1 domain mediates binding to protein aggregates, while ClpB is recruited via its M-domain to Hsp70 coating the aggregate surface. **(B)** Contributions of ClpB and ClpG to protein disaggregation during diverse heat stress regimes. The degree of protein unfolding depends on the absolute heat shock temperature. Extreme temperatures (>55°C) cause more complete protein unfolding leading to the formation of tight protein aggregates that are more difficult to be solubilized. ClpG is a more powerful disaggregase and exhibits robust disaggregation activity toward tight aggregates in contrast to ClpB/Hsp70. ClpB and Hsp70 (DnaK) unfold during extreme heat shock leaving ClpG as only functional disaggregase in cells.

The mechanistic differences between the ClpB and ClpG disaggregases are reflected in their domain organizations. ClpG harbors an additional, unique N-terminal domain (N1) that mediates the direct binding to protein aggregates (Figure 4A) thereby bypassing the requirement for a targeting factor (Lee et al., 2018; Katikaridis et al., 2021). Transplanting the ClpG N1-domain onto ClpB enables for autonomous aggregate binding and high disaggregation activity upon additional abolition of M-domain repression (Katikaridis et al., 2021). The Hsp70-independent activity of ClpG also demands for a different regulatory mode controlling its ATPase and threading activities. ClpG harbors an M-domain of reduced size that will not allow to form a repressing belt around the ATPase ring as observed for ClpB. This explains why ClpG activity control does not involve a partner protein. The two N-terminal domains N1 and N2 were recently shown to repress ClpG ATPase activity as their deletions lead to high ATP hydrolysis rates that were comparable to fully activated ClpB (Katikaridis et al., 2021). The functions of Hsp100 NTDs in substrate binding provides a potential path for ClpG regulation via substrate binding to N1 and N2 domains. Indeed, a peptide substrate that interacts with the N1 domain causes full activation of ClpG ATPase activity (Katikaridis et al., 2021). In this most simple scenario of ClpG activity control, high ATPase and threading activities will be also restricted to the surface of protein aggregates as shown before for ClpB. How the N1 domain provides selectivity for protein aggregates is currently unclear. Similarly, the allosteric pathways triggering ClpG ATPase activity upon aggregate binding remain to be determined.

## Interplay Between ClpB and ClpG Disaggregation Machineries

The ClpG disaggregase is currently present in selected Gram-negative bacteria and always coexists with the canonical ClpB/Hsp70 disaggregation system. This raises the question whether the two disaggregases cooperate. There is so far no evidence that the two systems work synergistically but they rather act independently. *In vitro* Hsp70 can inhibit ClpG disaggregation activity as they both compete for binding to protein aggregates (Lee et al., 2018). The two disaggregation systems therefore function as parallel activities in the solubilization of aggregated proteins. Accordingly, deleting both disaggregation systems in *P. aeruginosa* led to most severe loss of heat resistance as compared to single knockouts (Lee et al., 2018). Similarly, expression of *clpG* can restore heat resistance and protein disaggregation in *E. coli* MC4100 *dnaK* and *clpB* mutants (Lee et al., 2018; Kamal et al., 2021).

Since both disaggregation systems compete for binding to protein aggregates their contributions to cellular disaggregation activity will depend on their total levels. In *P. aeruginosa* ClpG levels are dependent on the growth phase. They are low during logarithmic growth and strongly increase during stationary phase. Accordingly the contribution of ClpG to heat resistance is high in the latter growth phase, while ClpB/Hsp70 activity is dominating during the logarithmic phase. Notably, *clpG* expression is not enhanced upon heat shock in contrast to *clpB* (Lee et al., 2018; Ma et al., 2020). The high expression of *clpG* during stationary phase might reflect a cellular strategy to prepare for future stress conditions circumventing the inability of cells to trigger stress responses during extreme heat shock. The increased production of ClpG during this growth phase might also help bacteria to cope with protein aggregation that is induced upon prolonged stationary phase (Pu et al., 2019). ClpG-mediated protein disaggregation might thereby aid faster re-growth once nutrients are again available.

As pointed out above ClpB and ClpG disaggregases can functionally replace each other, however, this only holds true in a certain temperature range. More severe heat stress conditions increase the dependence of bacterial survival on ClpG activity, consistent with the genetic link between extreme heat resistance and *clpG* presence. Why does ClpG but not ClpB provide superior heat resistance to bacteria? *In vitro* the disaggregation efficiency of ClpB/Hsp70 declines when the denaturation temperatures of thermolabile model substrates are increased (Katikaridis et al., 2019). These high temperatures likely enhance the degree of protein unfolding and thereby increase the number of interactions between unfolded proteins upon aggregation (Figure 4B). In consequence a higher threading force applied by an Hsp100 disaggregase will be required to break these interactions and extract the misfolded proteins from the aggregate. As described before, the threading power of the ClpB/DnaK system is limited, rationalizing why its disaggregation activity is low toward tight protein aggregates. In contrast, ClpG disaggregation activity is hardly affected by the heat stress regime applied to unfold and aggregate model substrates and stays robust over a wide temperature range *in vitro* and *in vivo* (Katikaridis et al., 2019). This is explained by a high unfolding activity of ClpG, enabling the disaggregase to unfold the tightly folded YFP moiety of an aggregated Luciferase-YFP fusion protein. Notably, *clpG* expression even at high levels is not linked to cellular toxicity despite its high unfolding power (Katikaridis et al., 2021). This is different from activated ClpB M-domain mutants, suggesting a more stringent substrate selection by ClpG, which likely only targets protein aggregates while avoiding interaction with other proteins like, e.g., nascent polypeptide chains.

Another, fundamental difference between the two disaggregation systems was recently revealed when determining their thermal stabilities. *E. coli* ClpB and DnaK have melting temperatures (*T*_M_-values) of approx. 60°C (Palleros et al., 1992; Kamal et al., 2021). Short temperature pulses (65°C) as applied during thermization will therefore lead to unfolding of ClpB and DnaK, entirely eradicating the ability of cells to revert protein aggregation if they only encode for the canonical disaggregation system (Figure 4B). In contrast, *P. aeruginosa* and *E. coli* ClpG are more stable (*T*_M_: 70°C), enabling the disaggregase to withstand extreme heat stress regimes. Thus under thermization conditions ClpG will remain the only functional disaggregation machinery, providing another rationale why only ClpG confers extreme heat resistance (Figure 4B). Notably, the determined *T*_M_ value of ClpG correlates with the upper limit of temperature resistance (70°C) provided by the LHR cluster (Ma et al., 2020).

## Protein Disaggregases as Drug Target

Bacterial protein disaggregases are not essential for viability, neither during normal growth conditions nor during mild, physiological heat stress. Therefore they have not been considered as attractive drug target so far. This view has now changed due to the established link between protein aggregation and bacterial dormancy and the emergence of the superior disaggregase ClpG.

Protein aggregation is linked to bacterial dormancy and the formation of persister cells, which are insensitive to antibiotics. While protein aggregation can lead to bacterial survival of antibiotic treatment, ClpB/DnaK-mediated protein disaggregation seems required for outgrowth of dormant bacteria (Pu et al., 2019). Inhibiting the canonical disaggregase can thereby prevent proliferation of persisters upon stopping antibiotic treatment. The inhibition might additionally intensify their dormant state leading to a viable but non-culturable (VBNC) state that is incapable of resuming growth.

The loss of essential proteins by aggregation upon severe heat shock causes bacterial cell death. This causal link is used by temperature-based sterilization protocols avoiding bacterial contaminations in food production and of medical equipment. The spreading of the superior disaggregase ClpG represents a threat to these established procedures as it enables bacteria to withstand the applied stress conditions. Inhibiting ClpG disaggregation activity by small molecules will re-sensitize bacteria toward heat-based sterilization and massively reduce contaminations.

Next to inhibiting protein disaggregases, their drug-induced allosteric activation could also be employed as anti-bacterial strategy. Activated Hsp104/ClpB M-domain mutants exert severe cellular toxicity (Schirmer et al., 2004; Oguchi et al., 2012; Lipinska et al., 2013) that is caused by their uncontrolled protein unfolding activities. Inducing a persistently active disaggregase by small molecules therefore represents an attractive alternative strategy. This strategy seems already adopted in nature as the natural antibiotic cyclomarin A (CymA) efficiently kills *Mycobacterium tuberculosis* by targeting the Hsp100 member ClpC1 (Schmitt et al., 2011). CymA binding causes persistent ClpC1 activation and leads to uncontrolled protein degradation by the ClpC1/ClpP1/2 protease (Maurer et al., 2019).

## Bacteria Lacking ClpB and ClpG: Existence of Potential Alternative Disaggregation Machineries

While ClpB exists in most bacteria there are Gram-positive species that do neither encode for ClpB nor ClpG raising the question which cellular machinery functions as disaggregase. In the model organism *Bacillus subtilis* there is solid evidence that this activity is executed by the Hsp100/AAA+ member ClpC. *B. subtilis clpC* mutant cells exhibit a heat-sensitive phenotype (Kruger et al., 1994; Msadek et al., 1994) and they are largely impaired in protein disaggregation (Hantke et al., 2018). ClpC localizes to stress-induced protein aggregates (Kruger et al., 2000; Kirstein et al., 2008), further supporting a role in protein disaggregation. Indeed, ClpC exhibits solid disaggregation activity *in vitro* (Schlothauer et al., 2003). Here, ClpC relies on cooperation with the MecA adaptor protein, which resembles the role of Hsp70 for ClpB by targeting substrates to ClpC and simultaneously stimulating ClpC ATPase activity. *B. subtilis ΔmecA* mutants are not stress-sensitive in contrast to Δ*clpC* cells (Schlothauer et al., 2003). This raises the question which adaptor protein assists ClpC disaggregation function *in vivo*. While ClpC and its partnering adaptor proteins show various mechanistic similarities to the ClpB/Hsp70 disaggregation system, there is also a fundamental difference: ClpC associates with the peptidase ClpP to form a bacterial proteasome. Such a disaggregating complex would exclusively degrade aggregated proteins, conflicting with the model that heat resistance relies on the rescue of the lost proteins. It will be therefore crucial to test whether ClpC can function independent of ClpP in protein disaggregation. Supporting such scenario, the *E. coli* Hsp100/AAA+ member ClpX, which functions together with ClpP in regulatory proteolysis, can also exert ClpP-independent functions *in vivo* (Jones et al., 1998).

Next to ClpC other Hsp100/AAA+ members might also play a role in protein disaggregation in Gram-positive bacteria. For instance *B. subtilis* ClpE is localizing to protein aggregates and aggregate removal is delayed in Δ*clpE* cells (Miethke et al., 2006). *Streptococcus pneumoniae* ClpL exhibits limited disaggregation activity *in vitro* (Park et al., 2015). Further studies are required to determine whether bacteria employ additional disaggregase to resist the heat.

## Author Contributions

PK, VB, and AM wrote the manuscript. All authors contributed to the article and approved the submitted version.

## Conflict of Interest

The authors declare that the research was conducted in the absence of any commercial or financial relationships that could be construed as a potential conflict of interest.
